# The Nuisance Created by Rendering Tanks and Manufacture of Fertilizers

**Published:** 1877-12

**Authors:** Oscar C. DeWolf

**Affiliations:** Commissioner of Health; City Hall, Chicago


### THE NUISANCE CREATED BY RENDERING TANKS AND MANUFACTURE OF FERTILIZERS.

While in Boston a few weeks ago the inquiry was made of me by a gentleman, and with much levity, if Chicago still regaled her citizens "and the stranger within her gates" with the delightful odors borne on the breezes from Bridgeport and the stock yards. To the average citizen of Chicago, levity associated with such an inquiry would be classed with that which good old Parson Kennedy, of Connecticut, termed the "damning levity of sin."

To the stranger it may seem a very ridiculous thing that a great city of 500.000 inhabitants with commercial interests and relations second to few, and a business sagacity, enterprise and energy, second to no city in the world; with a municipal organization "honest, capable and faithful" and with all its legal machinery intact, should suffer itself to be outraged for twelve years by one of the most intolerable nuisances that ever afflicted a people; but the citizen will recall the fact that a great industrial interest in the hands of a compact, able directory, and backed by a capital of more than $20,000.000 is not very ready to listen to the complaints of one or many individuals, and particularly if any effort to change the old and established order of things, will possibly exhibit itself on the wrong side of the ledger. And he will recall the other fact, that attacks upon it have seldom been organized, methodical and persistent, but rather fitful and frantic. Since it can be demonstrated that a process of rendering animal matter and manufacture of fertilizers can be conducted in a perfectly inoffensive manner, the time has come when the energies of those engaged in the business, should be expended in another direction than constructing tall chimneys in order to diffuse their fearfully offensive odors to a greater distance, and when summoned to appear in court, presenting themselves with their troup of laborers and retainers to swear that the premises are sweet as banks of flowers in June. The time has come when the citizen should regard it as one of the highest political duties, and a paramount social obligation, to insist that the issue should be squarely made and the following declaration of faith and work accepted by all, either the business referred to must be conducted in an inoffensive manner, or the business must move, or the city of Chicago must move.

There are gratifying indications that the more intelligent and honorable men engaged in the work, are disposed to co-operate with the authorities in an effort to remove the cause of complaint.

It is the purpose of this paper to show the amount of material rendered and manufactured into fertilizers within the limits of the city, and within one mile of its limits; the source and character of the offensive gases generated, and the methods by which a suppression of this great evil may be accomplished. There was slaughtered last year within the limits before mentioned

| | | |
|---|---|---|
| 299,020 | - - - | Beef Cattle, |
| 3,058,444 | - - . | Hogs, |
| 168,170 | - - - | Sheep. |

Each beef is estimated to yield 50lbs. of offal, the hog 20lbs. and the sheep 7lbs.; a total of 77,277,070lbs. of animal matter for the rendering tanks. This will include head and feet, but not the blood, of which there were 224,500 barrels of 32 gallons each. In addition to this we must bring the odds and ends, bones, scraps, etc., from all the markets, the decayed fish, and the beef found in the markets and slaughtering houses and condemned by the sanitary inspectors as unfit for food—of which there has been condemned and thrown into the rendering tanks, from April last to this date, 126,400lbs.

In the disposition of this immense amount of animal matter often in all stages of decomposition—and the animal tissues containing fat are much more subject to rapid decomposition than other portions of the animal—the first process is to extract the tallow and fat from the mass and thoroughly separate the soft tissues from the bone. This is accomplished in steam-tight, iron tanks, of from 8 to 10 tons capacity, into which the crude material is thrown and subjected to a cooking for several hours by a steam pressure of from 40 to 60lbs. to the sq. inch.

The slaughtering and packing houses of our neighborhood have an aggregate of 237 tanks as above described.

It is during this exposure to great heat within the tanks that the foul gases are generated, and if conducted directly within the flue from the tanks, it is practically impossible to construct a chimney so high that the gaseous and noxious effluvia will not diffuse themselves, to cool and descend upon the surrounding neighborhood.

These gaseous products of heat and decomposition are the various sulphur combinations, sulphuretted hydrogen, phosphuretted hydrogen, and ammoniacal gases, and they may be more or less offensive, depending upon the degree of decomposition taken place in the raw material before the cooking has begun. Nevertheless, under any circumstances and no matter how fresh the materials, there will be given off offensive odors during the melting process under such great heat from the nitrogenized matters always in combination with such material.

Again, if the freshest fats are subjected to a heat of 500° Fahrenheit, they begin to undergo a decomposition; the base glycerine is decomposed and a product called acroleine—an exceedingly acrid and irritating vapor—is set free. Methods have been proposed by science for the extraction of these fats from the raw material by means of the hydro-carbon oils, and by naphtha; also, by using bi-sulphide of carbon, in which the yield is much greater and the product said to be superior. These are patented but not well understood in this country, although extensively applied in Germany and France (Moreau Morris). The attempts made to destroy these gases by "condensing," washing, burning, etc., etc., have been numerous, but rarely scientific, and not largely successful as this community will testify. The old and rude process of the open kettle, with its unrestricted escape of steam and gases, is more satisfactory than the modern steam-tight, boiler-iron tank with its escape-pipe leading directly into a chimney 200 feet high, for in the one case the gases are left nearer the ground and localized, while in the other they may be carried by the winds for miles before sufficiently cooled to fall. That these gases cannot be destroyed by simply conducting them into or under an ordinary furnace fire of from 500 to 800 degrees of heat, will be understood by any one who comprehends their nature and composition, yet this has been the usual method pursued in our vicinity. The sulphur compounds are undoubtedly destroyed by combustion, but the ammonia with all the fetid products held in association with it, pass unchanged up the flue. The process of "condensing," for which much is said and claimed by those interested in the operation, is equally fallacious, for by passing the gaseous products evolved from the rendering tanks through large volumes of cold water, nothing is effected except the condensation of the steam into water, leaving the gases dry—but ammonia will not burn, wet or dry, without special apparatus for its combustion.

There are two methods in practical operation to-day by which these offensive products are satisfactorily destroyed. The first was patented by Lockwood & Everett, of New York, in 1865, and is now in successful practice there. Steam-tight iron tanks are used for the reception of the offal, and these tanks are heated by steam applied upon the outside only.

The offensive gases are forced by the pressure evolved within the tanks, through an iron pipe, which passes over them and is connected with each tank by a short connecting pipe, into a system of iron coils which are placed in a separate and independent furnace ; here they are subjected to a heat of from 1500 to 1700 degrees Fahrenheit, and thus superheated, they are brought by an ingenious and scientific arrangement of argand burners in contact with the flame of a furnace in combination with sufficient oxygen to produce a perfect combustion and without a trace of offensive odor remaining.

The second process which I shall describe was the experiment of Mr. James Turner, of this city, which resulted in Turner's patent (1872), which has been modified and patented by Mr. Dennis Wood, also of this city. The process is essentially the same in both patents, the modification consisting of a different apparatus for condensing the steam. The same steam-tight tanks and conducting pipes are used as in the New York patent, but by our process heat is applied to the mass by carrying the steam within the tanks. The gases commingled with the steam are forced through the conducting pipe into the bottom of a large tank of cold water, which is constantly renewed ; the gases rise through this body of cold water, the steam is condensed, and the dry gases collect in the upper chamber of the tank. From this chamber they are conducted by a pipe into the bottom of an iron tank partially filled with gasoline or other of the lighter distillates of coal tar. As the gases rise through this highly inflammable material they become charged with it to such a degree that when conducted under an ordinary furnace they burn with fierce intensity.

When thus charged, they may be conducted over the building and used for illuminating purposes, as is done at Mr. Turner's establishment.

By either of the processes referred to, the noxious gases from rendering tanks may be entirely destroyed.

There remains still another source of great offence. When the contents of the tanks are sufficiently cooked, the steam is shut off, and when cooled down the material is drawn into large wooden vats at the base of the tanks. As they cool the fats are skimmed from the surface, and the remaining material is removed in carts to the houses for the manufacture of fertilizers. This process—the conversion of the tank material into a commercial fertilizer—is simply driving off the water from the "tankings" to about 13 per cent., and grinding the dried product.

The drying machine generally consists of a revolving cylinder, thirty feet long and three feet in diameter, inside of which are arms for the purpose of comminuting and breaking up the material. The heat is supplied by fires underneath, and in some instances a current of hot air passing through the cylinder.

When in position that end is highest where the material is fed into the dryer; the refuse constantly revolving and agitated by the projecting arms, is kept in motion until it passes out at the other end in a dry state.

From two to ten tons can be passed through in an hour. (Ben. C. Miller, paper read before Public Health Association, Philadelphia.) In these houses also the blood is received in barrels from the slaughtering houses, and after being treated with sulphuric acid (to fix the ammonia in the stable form of sulphate of ammonia), it is passed through the dryer in the same manner as the tankings. Passing through such intense heat and drying so rapidly, the volume of steam and gases evolved are so enormous that the various processes in use for condensing or burning, have proved entirely inadequate and unsatisfactory.

It is probably true that destruction of these gases by chemical decomposition is the only practical method of dealing with them. As a tentative measure in this direction, the following means have been devised. It was found, by experiment in a small way, that by passing these gases through a solution of per-nitrate of iron, or over surfaces covered with this salt, the offensive odors were entirely destroyed. The problem to be solved, then, was simply this: by what means can the large volume of gases from the drying cylinders be brought into contact with this salt so intimately as to be completely decomposed?

At the house of Messrs. Oberndorf & Shepherd, (stock yards) a matched plank, steam-tight box, was built 20 feet long by 10 feet broad, and 10 feet deep. Ten matched horizontal partitions were built within the box, one foot apart, and each partition four inches short at alternate ends. Each partition, from its attached to its free end, has a fall of a half inch. The spaces between the partitions were stuffed full of sheet iron scraps, amongst which was placed in each space from 15 to 20 lbs. of nitrate of potassa.

Entering the lowest space at the end of the box is a conducting pipe, two inches in diameter, from a little sheet iron stove or furnace near by, in which brimstone is burned. The gases from the drying cylinder are drawn into a pipe 18 inches in diameter by an iron fan, making 2,000 revolutious a minute, and this pipe enters the box in the same space as the pipe from the brimstone furnace. The gases after passing backwards and forwards between the partitions, escape from the upper space through a short pipe, with all offensive odors destroyed.

This box has been in operation about 60 working days, and it is found that 40 lbs. of brimstone, costing $1.30, will destroy all that is offensive in the drying of 20 tons of fertilizer, which is the average day's work at this house, when running.

The modus operandi is as follows: The sulphurous acid, generated by the combustion of brimstone, receives the equivalent of oxygen necessary to convert it into sulphuric acid, and acting slowly, but with sufficient energy, upon the nitrate of potassa to chemically decompose it and set free nitric acid fumes. These fumes, coming in contact with the iron through which they pass in all directions, form the per-nitrate of iron, which coats each surface of every iron scrap; any excess of the iron salt is taken into solution by the steam which comes in from the dryer, and this solution slowly passes along each partition, dripping from the free end of the upper one to that below, and along that to the next, and so on to the bottom, where it is collected in a vessel to be used as a disinfectant about the premises.

It will be observed that the gases entering the box below must pass not only over 2,000 feet of board surface, covered with the solution of the iron salt, but also over the immensely multiplied surface which each iron scrap presents.

The cost of the box filled and ready to work is less than $150, and I infer from the work so far that the box will not require to be refilled oftener than every four months. This can be easily done by removing one end, and at a cost of about $30. It only remains to report the result of the years work in attempting to suppress this great nuisance.

On the first of January, 1877, there were only 8 tanks connected with an efficient apparatus, to destroy their gases, to-wit:

| | |
|---|---|
| Mr. James Turner, - - - | 4 tanks. |
| Mr. Dennis Ward, - - | 4 " |

The remaining 229 tanks, when in operation, were pouring their noxious odors sometimes directly into the out-of-doors, sometimes under the furnace, to escape at the top of the chimney, sometimes into a tank of water to be "condensed," and from there to the flue and out; but whichever way they passed the result was the same—an intolerable stench "going with the wind."

The following houses have recently connected their tanks with a perfectly satisfactory apparatus:

| | |
|---|---|
| Turner Bros., - - - - | 2 tanks. |
| Shönemann & Co., - - | 5 " |
| O'Malley & Sons, - - - | 4 " |
| J. L. Hancock, - - - | 6 " |
| J. E. Smith, - - - - | 4 " |
| J. Flanigan, - - - | 2 " |
| E. D. Chapin, - - - - | 4 " |

The following houses are at this date putting in, and by Dec. 1st will have completed the same apparatus:

| | |
|---|---|
| Fowler Bros., - - - - | 10 tanks. |
| Hutchinson, - - - | 35 " |
| Higgins, - - - - | 15 " |
| Bottsford, - - - - | 8 " |
| B. F. Murphy, - - - | 15 " |

Making a total of 118 tanks in operation without offense.

There are 53 tanks in houses at present closed, and 66 tanks in houses, the proprietors of which have up to this time refused or neglected to correct their evil work. "No business detrimental to the public health, and which is carried on in defiance of great sanitary laws, positively neglecting to adopt the remedies at hand, should be tolerated in civilized communities."

Oscar C. DeWolf, M.D.,
Commissioner of Health.

City Hall, Chicago, Nov. 15th, 1877.

